# The addition of a flange does not improve the pressure generated during cemented acetabular cup implantation

**DOI:** 10.1002/jbm.b.35077

**Published:** 2022-06-03

**Authors:** Alexander T. Boote, David J. Deehan, Kenneth S. Rankin, David C. Swailes, Philip J. Hyde

**Affiliations:** ^1^ School of Engineering Newcastle University, NE1 7RU Newcastle upon Tyne UK; ^2^ Freeman Hospital Newcastle Upon Tyne UK

**Keywords:** acetabular pressurization, flanged acetabular cup, PMMA bone cement, total hip arthroplasty, total joint replacement

## Abstract

Flanged acetabular cups were developed with the rationale that, at insertion, they would increase the pressure of the cement and improve penetration of cement into the acetabular bone. Various studies have been inconclusive regarding their effectiveness. In this work, we aimed to eliminate all confounding factors and measure the pressures generated during acetabular pressurization and cup implantation using a simplified steel acetabulum, high precision pressure transducers, proper surgical techniques and two acetabular cups, identical apart from the addition of a flange to one. It was found that the flanged acetabular component did not significantly increase the pressure in the acetabulum and in some cases reduced the pressures generated when compared to an unflanged cup. The addition of a flange did not reduce the pressure differential between the pole and the rim of the acetabulum, nor did it have a significant effect on pressure lost over the cup implantation period. It was concluded that flanged acetabular cups provide no significant improvement in the pressures generated in the acetabulum during acetabular cup implantation. It is hypothesized that the flange may be seen as a design feature intended to slow the insertion of the cup into the cement, thus requiring the surgeon to apply a larger load in order to correctly position the acetabular cup; in this way larger pressure will be generated.

## 1 INTRODUCTION

**FIGURE 1:**
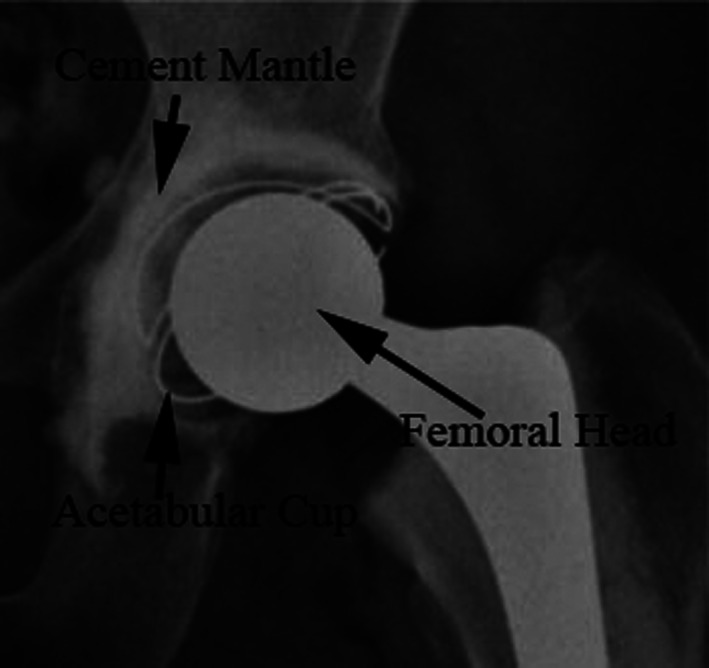
A radiograph of a cemented THR with annotations.^1^

Total hip replacement (THR) involves the implantation of a new acetabular cup in the acetabulum and a new femoral head onto the femur (Figure 1). Although a cementless fixation method for acetabular cups has become the most popular form of fixation in the United Kingdom, cemented fixation still remains the gold standard method of fixation for metal on polymer and ceramic on polymer hips due to its superior longevity and relatively low cost.^2^, ^3^ In cemented THRs, the new acetabular component is held in place with bone cement which stabilizes it within the acetabulum. The cement mantle between the new cup and the bone of the socket acts as a grout to stabilize the cup with immediate mechanical fixation to the bone by interdigitation. This is the process that cement pressurization achieves: the fluid cement is pressurized to flow into the trabecula bone voids and form small fingers (digits) of cement which resist shear forces which would otherwise try to rotate the cup in daily living. Therefore, pressurization of bone cement is crucial for both immediate postoperative and long‐term cup stability. Commercial polymethylmethacrylate (PMMA) bone cement comes as a powder and liquid which, when mixed, starts a polymerization process that begins at a relatively low viscosity and progresses to a dough‐like substance (at which time implantation begins) and finishes as a solid acrylic polymer. During the operation, the surgeon removes the articular cartilage and cortical bone of the acetabulum though reaming, exposing the porous cancellous bone beneath. The cement is then mixed and inserted into the cavity. A device consisting of a silicone spherical cap, usually called a pressuriser, is used to seal the acetabulum and force the cement into the bone matrix. Once the cement is sufficiently pressurized the pressuriser is removed and the acetabular cup is placed into the still doughy cement, and a force is applied again until the cup is correctly positioned. Prevalence of early loosing of the acetabular cup show that innovations in cementation techniques are still necessary.^2^

Of all failed THRs, many fail due to aseptic loosening of the cup.^2^, ^4^ Early radiolucent lines on the radiograph between the cement and the bone, particularly progressive ones, are a reliable predictor of later loosening.^5^, ^6^, ^7^, ^8^ The reamed acetabulum is a shallow cavity with a large, irregular opening; this makes it difficult to maintain a high pressure at the bone surface both during pressurization and cup insertion. The addition of a flange to the acetabular cup was claimed to improve pressurization, prevent the acetabular cup bottoming out (where the cup makes contact with the bone, thus stopping further pressurization) and to minimize cup movement during implantation.^9^ A flange was proposed to provide uniform pressurization and thereby optimize cement intrusion to the subchondral bone which has been showed to improve the interface strength.^10^

The literature on this topic contains limited data regarding the pressures generated at the acetabulum surface and there is contradictory experimental evidence regarding the efficacy of flanged acetabular components.^11^, ^12^, ^13^, ^14^, ^15^, ^16^, ^17^ Therefore the aim of this study was to contribute to the existing literature regarding whether the addition of a flange to the acetabular component alters the cement pressure distribution at the surface of a model acetabulum during cup implantation. It also aimed to provide a detailed pressure profile at the acetabulum surface which all other similar studies fail to provide.

Three key questions were asked:

Does the addition of a flange to the acetabular component increase the pressure of the cement at the cement‐bone interface?

Does the addition of a flange to the acetabular component affect the pressure distribution at the cement‐bone interface?

Is pressure maintained throughout pressurisation and cup implantation and does the addition of a flange significantly affect this?

## 2 MATERIALS

**FIGURE 2:**
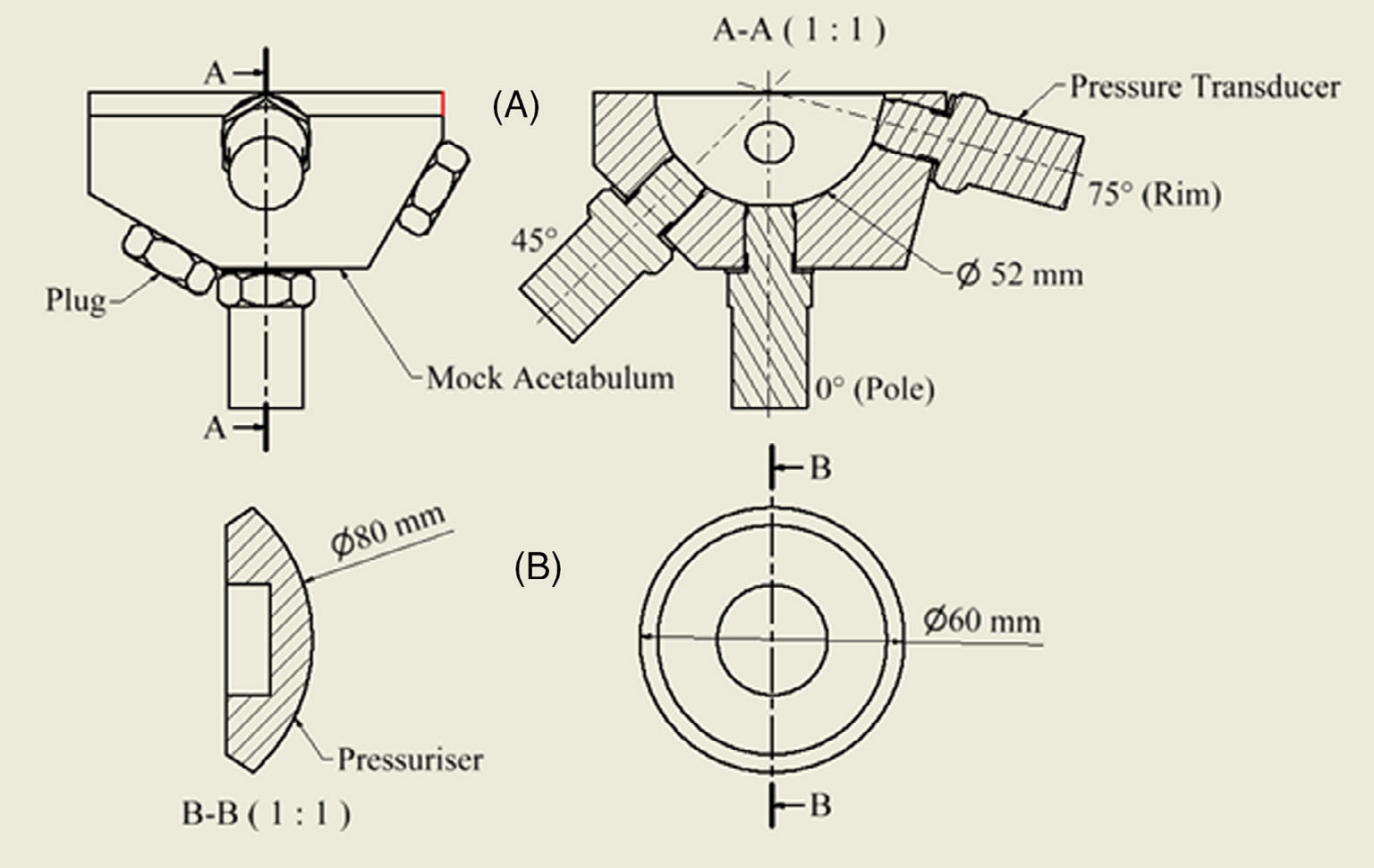
Engineering drawings with all relevant dimensions of the mock acetabulum (A) and the Depuy Smartseal pressuriser (B)

An acetabulum model was manufactured from stainless steel 304 with a 52 mm hemispherical bore, a diameter to which the acetabulum is often reamed in vivo. Steel was selected as it would provide a very accurate surface for the pressure transducers to lay flush on, a porous model would closer represent the surface texture of the acetabulum; however, the cement should only contact the surface flush with the acetabulum, this would be impossible using a porous model; previous studies use a rubber glove to separate the pressure transducer from the cement but this would invalidate the pressures recorded.^18^ The diameter was confirmed to be within 0.01 mm of the expected value using a coordinate measuring machine. The model included tapped holes for pressure transducers at 0° (pole), 45°, and 75° (rim) from the direction of forcing (Figure 2a).

A Hivac™ bowl (Summit Medical LTD, Gloucestershire) or a glass bowl and a PTFE spatula were used to mix the cement.

A Depuy Smartseal acetabular pressuriser (DePuy, UK) was used for pressurization of the cement. It was 80 mm in diameter and consists of a silicone hemispherical segment and is designed to seal off the acetabulum cavity with the cement still inside (Figure 2b, Figure 4).

**FIGURE 3:**
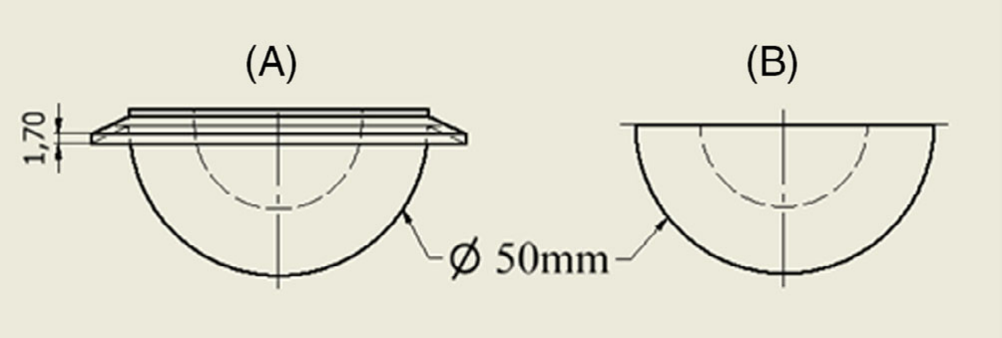
Otherwise identical flanged (A) and unflanged (B) acetabular cups

The acetabular cups were manufactured from HXLPE (highly‐crosslinked polyethylene). A flanged and an unflanged cup were designed so that the only difference between them was the flange. Both had an external diameter of 50 mm and an internal diameter of 28 mm, this would leave a cement mantle of around 1 mm thick if the centres of the cup and the acetabulum cavity were aligned. The flange had a thickness of 1.7 mm and diameter 63 mm (Figure 3).

Omega PX61V0 pressure transducers were used with an Omega TXDIN1600S bridge for amplification and data acquisition. The pressure sensors were calibrated using a loading program and a doughy substance which produced known pressures at each position of the acetabulum; this was done prior to each experiment session. The transducers were made flush to the acetabulum hemispherical surface using shim washers. The data was filtered using a first order, low pass Butterworth filter with a cut‐off frequency of 0.00625 Hz, selected using the Nyquist criterion.

A Type K thermocouple was used to monitor the temperature, as temperature is often used to monitor the progress of polymerization. The thermocouple was inserted into the acetabulum cavity between the acetabular rim and the pressuriser. The location of the tip of the thermocouple was not controlled; therefore, the magnitude of the temperatures measured cannot be directly compared between tests but the data can still be used to calculate the cure‐time which is defined as the time at which the cement was halfway between the ambient temperature and maximum temperature reached.^19^

CMW 2 (Depuy Synthes); a high‐viscosity cement frequently used for fixation of the acetabular component was used to implant the acetabular cups. For each experiment two 20 g packets of cement were used as this sufficiently filled the cavity. The cement used has been subject to many previous studies.

**FIGURE 4:**
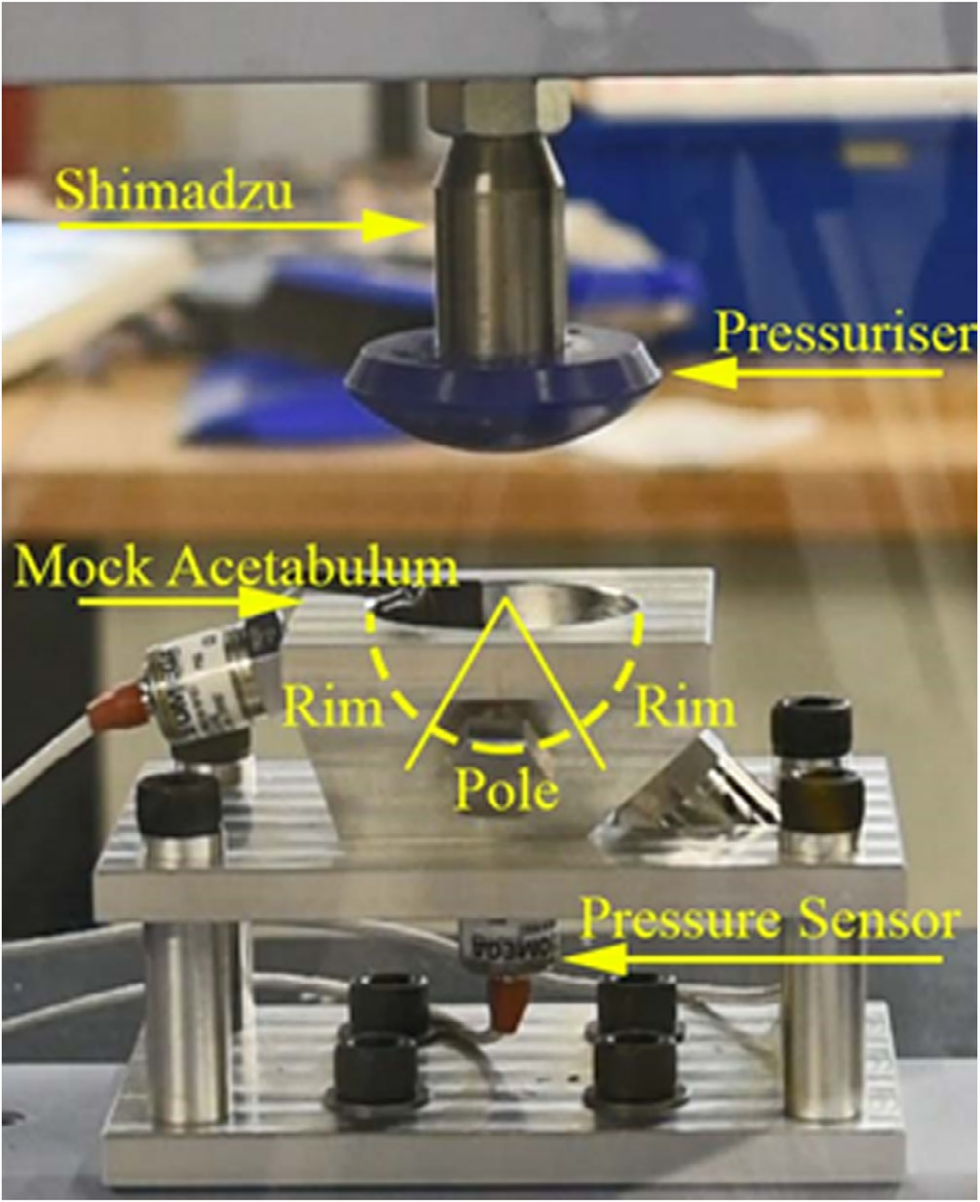
Mock acetabulum and Depuy pressuriser experimental set up showing the sensor positions in relation to the rim and pole.

The assembled rig was mounted into a Shimadzu ADS‐X which was used to apply load. It was fitted with a 1 kN load cell (Figure 4). Note that during surgery, the cup is implanted at 40° to the transverse plane, however, the force applied by the surgeon is orthogonal to the plane of the cup face, therefore the experimental set up here is the same.

All equipment used was manufactured with a tolerance of ± 0.05 mm, with consideration of the design of the rig, the loading was always applied within 0.25 mm from the center of the acetabulum cavity.

## 3 METHODOLOGY

**FIGURE 5:**
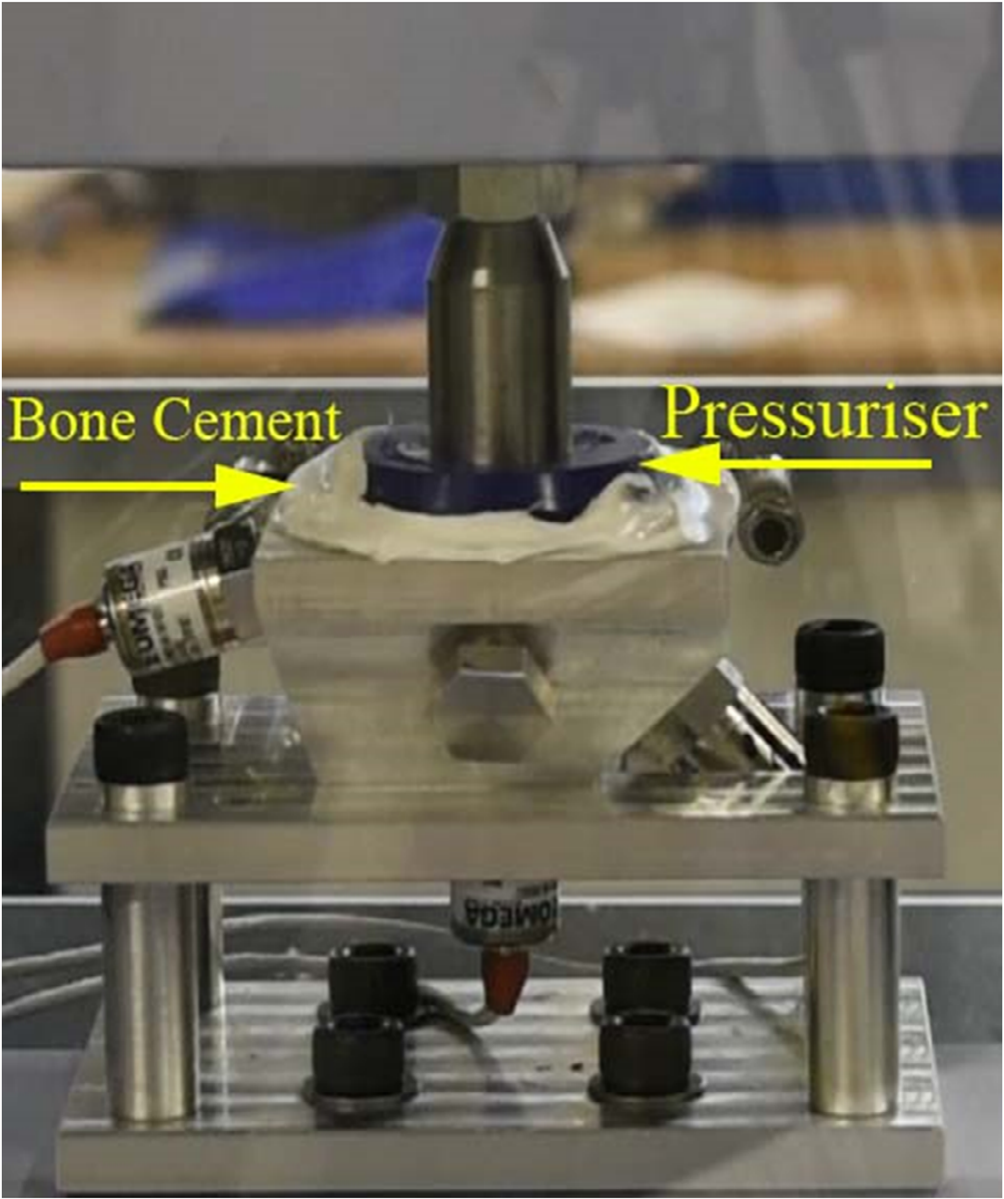
A force was applied to bone cement in acetabulum using 100 N force on Depuy pressuriser.

The methodology employed here is identical to that of a previously published study.^20^ The temperature of the laboratory was between 20.5°C and 23°C for all experiments. The humidity of the lab was between 45% and 50%. All equipment was left in the lab to ensure that the temperature of the equipment was static.^19^ Mold release spray (Silicone Mold Release Agent, Ambersil) was used to ensure that the cement mantle could be removed from the model acetabulum. The Shimadzu was force controlled with a maximum stroke rate of 40 mm/min. The PMMA powder and the MMA liquid were either mixed by hand in an open glass bowl with a polyethylene spatula at around 1 Hz until homogenous or in a Hivac™ bowl under a 0.4 bar vacuum at a similar frequency until homogenous. For both conditions the cement was then left to rest until the cement no longer adhered to surgical gloves (clinically defined as the dough point.^19^). The cement was then inserted into the acetabular cavity and the loading program for pressurization was started. The cement was pressurized for 100 s at 100 N (Figure 5). The load used during pressurization was determined from the literature. Bernowski et al. used a force of 210 N for acetabular pressurization.^21^ The pressures generated in an experiment by New et al. resulted in similar pressures at the acetabulum as was found in preliminary experiments using a force of 100 N.^22^ Parsch et al. used a force of 60 N for pressurization.^18^ Beverland et al. used a 10 kg mass to apply force during pressurization.^15^ A load of 100 N was used as it reflected the previous literature and the opinion of the surgeons co‐authoring this paper. The timing was determined so that pressurization and cup insertion would both be completed within the working phase of the cement.

**FIGURE 6:**
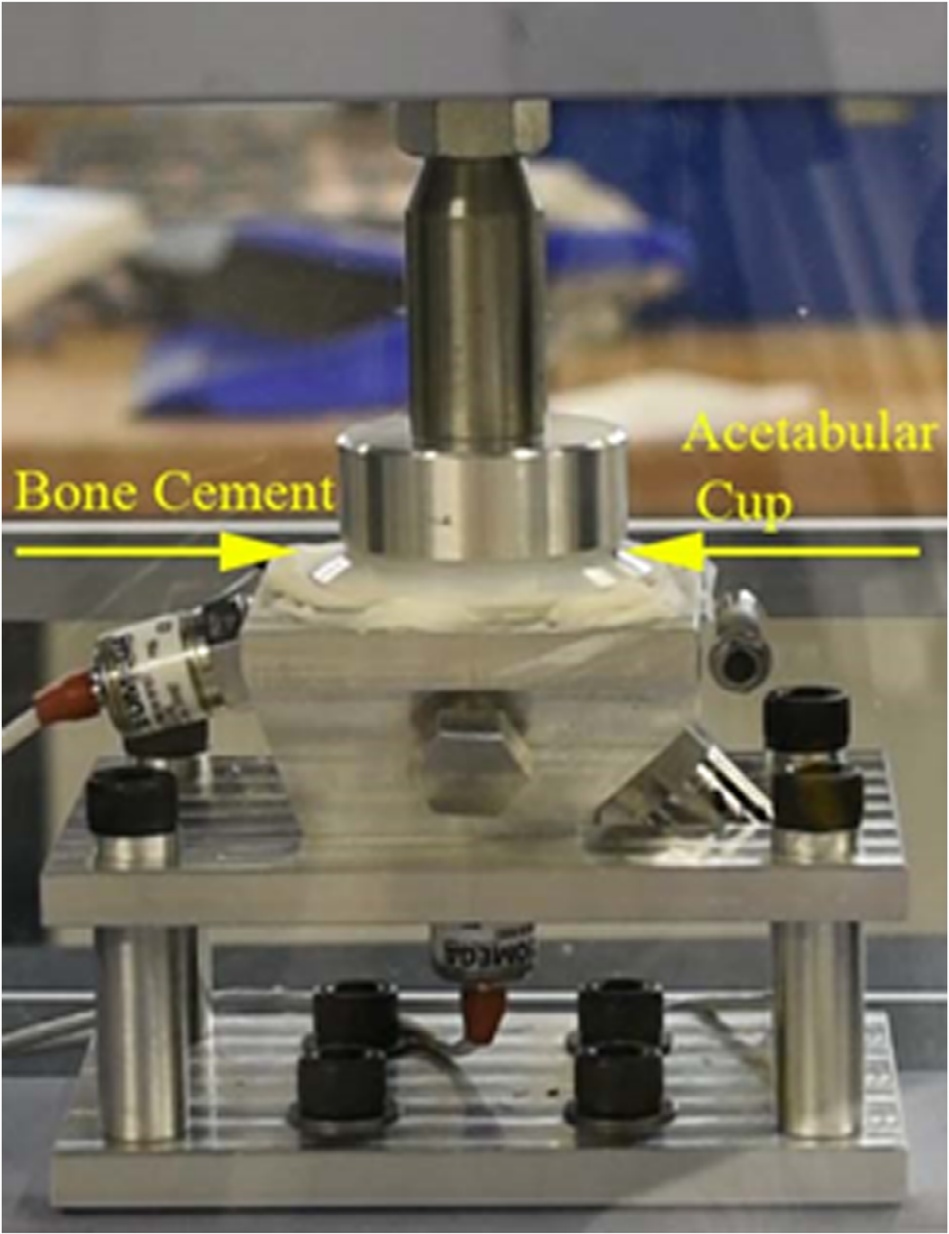
Pressure applied to the acetabular cup using a 50 N force.

The pressuriser was then removed from the Shimadzu and a cup was placed into the cement. The cup implantation program was started, a load of 50 N was applied until the cement was fully cured (Figure 6). After the cement had fully cured, the cement mantle was removed, and another test was performed. This was repeated five times for each of the four testing conditions: two cup designs and two mixing methodologies.

For this experiment, pressurization and cup insertion were performed within the working time advised by the cement manufacturer. Preliminary experiments were used to determine the cup insertion load. These preliminary experiments consisted of implanting flanged and unflanged acetabular cups with various loads ranging from 25 to 75 N. Larger loads caused the unflanged cup to bottom out and smaller loads meant that the flanged cup did not sufficiently enter the cement. Upon observation, a load of 50 N resulted in both cups being suitably inserted into the cavity whilst avoiding contact between the cup and the model acetabulum. Loads used in similar experiments were also around this figure. Although it is unclear, Ørskov et al. seemed to measure a load of 57 N for correct position of unflanged cups and 68 N for flanged cups.^23^ Bhattacharya et al. used a load of 70 N for both pressurization and cup insertion.^13^ Shelly and Wroblewski used an 8 kg weight for the application of load.^17^

**FIGURE 7:**
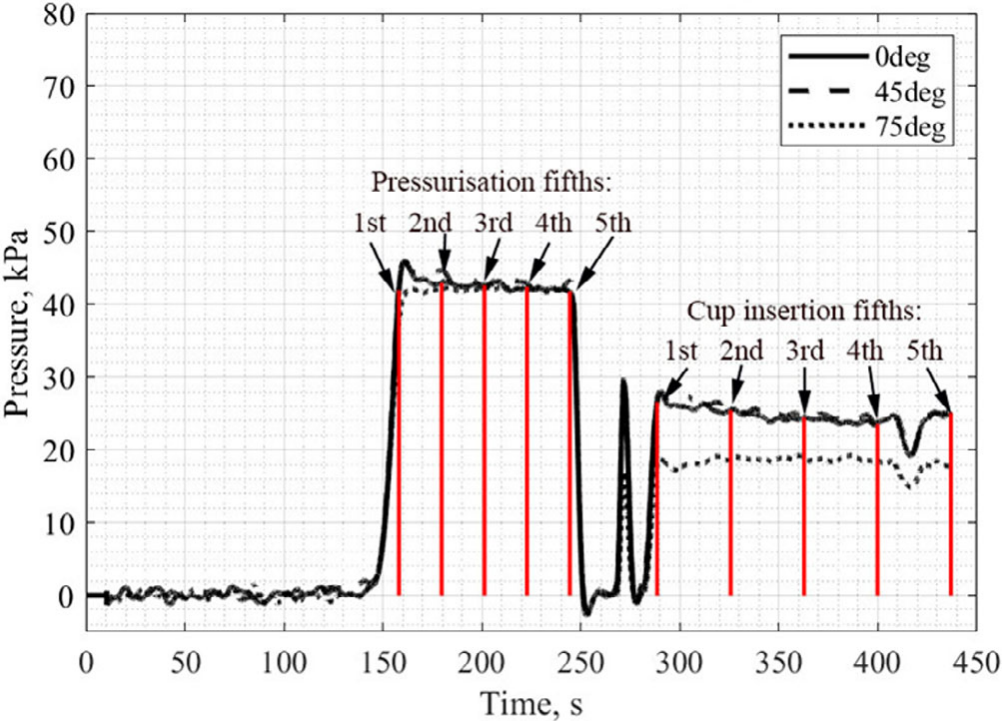
A typical plot with indication of how data are split up into fifths (pentiles) for further analysis.

The end of cup implantation was taken to be when there was a significant deviation from the average pressure. To allow a more detailed analysis of the continuous pressure curves they were divided into fifths and the pressure at each of these five points in time was taken and used for statistical comparisons. (Figure 7). This technique also allowed for analysis of how the pressure evolves, previous studies often only state the average or maximum pressure achieved during surgery, but this is not enough information for proper analysis.

A Ryan‐Joiner test was used to test for normality, if *p* ≤ 0.05 it was concluded that the pressure data at that time was not normally distributed. A student *t*‐test was used if both sets of data being compared were found to be normally distributed. If one or both sets of data were found to be non‐normal, then a Mann–Whitney test was used. A difference in the means was considered significant if *p* ≤ 0.05. This statistical methodology was used for all comparisons of data.

## 4 RESULTS

**FIGURE 8:**
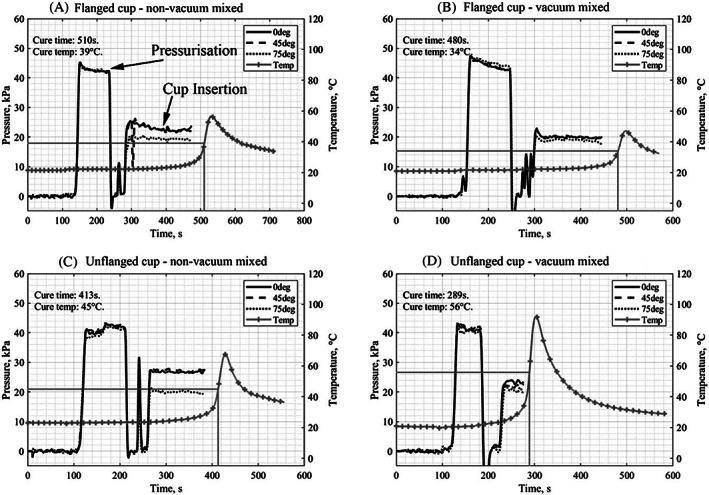
Four graphs showing a typical example of the pressure, temperature—time plot from each of the testing conditions. The pressure at various angles from the direction of forcing and the temperature through time are plotted. Pressurization and cup insertion are indicated in (A) and are in the similar positions in (b–d). The time and temperature of the cure point is also indicated

Typical annotated plots showing cement pressure and temperature over time for both vacuum mixed and non‐vacuum mixed flanged and an unflanged acetabular cup implantation can be seen below (Figure 8). Three pressure measurements were recorded at positions 0° (pole), 45° and 75° (rim). There were two key stages of the experiment: pressurization and cup insertion. The end of cup insertion always occurred near the cure time. After pressurization, when the pressuriser head was removed from the cement, a negative pressure can be seen; this is usually avoided in surgery through the surgeon twisting the pressuriser head before removal from the cement. The spike in pressure between pressurization and cup insertion is caused by the placement of the acetabular cup into the cement.

The averages and the standard deviations of the cement pressure for each pentile (fifth) of cup insertion, at each angle, for each condition can be seen in Table 1. The equivalent data for pressurization is not provided as no statistical difference was found in the pressures generated due to which cup was subsequently implanted (*p <* 0.05) and no significant change in pressure was observed through time (*p <* 0.05).

**TABLE 1:** A table containing the average pressures (and standard deviations) for each testing condition, at each angle from the direction of loading, at each pentile of cup insertion. Statistical differences between flanged and unflanged cups are indicated with ^a, b, and c^ indicating the relevant pair. The statistical test used for the results shown in italics was a Mann‐Whitney test due to non‐normal data.

Sample	Angle, °	1st, kPa	2nd, kPa	3rd, kPa	4th, kPa	5th, kPa
Flanged cup, non‐vacuum mixed.	0	24.16 (3.47)	25.74 (1.07)	24.78 (1.55)	^a^ *24.28 (1.65)*	25.93 (1.55)
	45	24.16 (3.30)	25.79 (1.40)	25.13 (1.78)	24.32 (1.98)	24.99 (2.30)
	75	18.75 (4.07)	20.70 (1.97)	20.46 (1.77)	20.02 (1.50)	19.44 (1.52)
Flanged cup, vacuum mixed	0	20.73 (3.80)	21.05 (1.74)	20.84 (1.68)	20.41 (2.06)	20.56 (2.58)
	45	20.52 (4.31)	20.82 (1.92)	20.65 (1.85)	^b^ *20.29 (1.64)*	^c^ *19.85 (2.09)*
	75	18.77 (3.66)	19.68 (1.92)	19.28 (1.27)	19.28 (1.55)	18.56 (1.67)
Unflanged cup, non‐vacuum mixed	0	25.70 (2.14)	26.61 (1.64)	26.80 (1.45)	^a^ *27.01 (1.79)*	27.85 (2.36)
	45	24.96 (2.41)	25.88 (1.59)	25.81 (2.08)	25.43 (2.05)	25.69 (2.90)
	75	19.67 (1.73)	21.08 (2.59)	21.41 (2.69)	21.73 (3.11)	21.76 (3.92)
Unflanged cup, vacuum mixed	0	22.64 (0.80)	22.37 (1.37)	21.98 (1.58)	22.71 (1.26)	22.74 (0.71)
	45	22.20 (1.09)	22.02 (1.20)	21.58 (1.53)	^b^ *22.45 (0.79)*	^c^ *22.85 (0.43)*
	75	20.78 (1.97)	20.41 (1.84)	19.79 (1.35)	20.00 (1.50)	19.29 (1.91)

*Note*: Statistical differences between flanged and unflanged cups are highlighted using a, b, and c to indicate the relevant pair.

The addition of a flange had little effect on the magnitude of pressure. There were only three significant differences in the pressures. Firstly, the unflanged cup generated larger pressures in the fourth pentile of cup insertion at 0° for non‐vacuum mixed cement than the flanged cup. The magnitude of this difference was 3.58 kPa, a percentage increase of 14.7% of the pressures generated for flanged cups. The second and the final difference in pressure was for the fourth and last pentiles of cup insertion for vacuum mixed cement at 45° from the direction of forcing. Once again, unflanged cups produced a larger force with a 1.81 kPa difference for the fourth pentile and 2.56 kPa for the final pentile, this is 8.76% and 12.6% increase of the pressure generated by the flanged cup.

**FIGURE 9:**
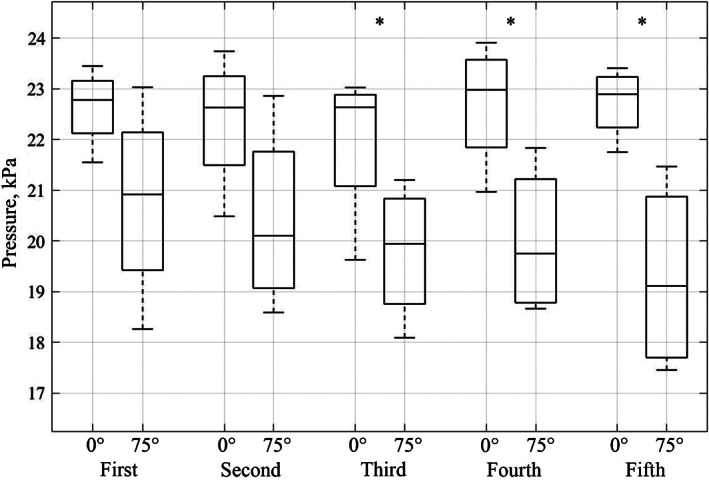
Boxplots of the average pressure for the unflanged, vacuum mixed condition. Each pair of boxplots represents the pressure at 0° and 75° for each pentile of cup insertion

With the exception of the first and second pentile of cup insertion for an unflanged cup with vacuum mixed cement, there was always a significant difference between the pressure generated at the rim and the pole of the acetabulum during cup insertion (Figure 9).

There was no statistically significant drop in pressure for any set of data.

Upon closer inspection of the removed cement mantles, it was found that none of the cups bottomed out and the cement mantle was thicker than 2 mm for all repeats at all angles from the direction of loading.

## 5 DISCUSSION

The aim of this study was to answer three questions regarding cement behavior in the acetabulum during implantation of unflanged and flanged acetabular cups. Firstly, it was found that the addition of a flange to the acetabular cup did not increase the pressure generated in the cement at the acetabulum bone surface during cup implantation. There were only three significant differences found in pressure due to the cup design; the unflanged cup generated larger pressures. Secondly, except for the first two pentiles of vacuum mixed, unflanged cup insertion; it was found that the pressure was always significantly larger at the pole of the acetabulum (0°) than at the rim (75°) during cup implantation; there is no evidence to suggest that the addition of a flange significantly reduced the pressure differential. Finally, it was found that there was no decrease in pressure over time for any of the testing conditions.

A good bond between the bone cement and bone is a key for the longevity of total hip arthroplasty implants as more interdigitation increases the contact area between cement and bone and thus decreases contact stresses.^10^ Suboptimal bonding can be observed on postoperative radiographs as a radiolucent line between the cement and the bone. These are most frequently observed near the rim of the interface.^24^ It has been shown that the penetration depth of bone cement into the bone is dependent on the pressure generated during implantation.^25^ The strength of the cement‐bone interface is dependent on the depth of penetration.^10^, ^26^ Therefore, it is key that the cement pressure generated during pressurization and cup insertion should be uniform and sufficiently large to achieve optimal penetration across the acetabulum.

### 5.1 Pressurization

In 1999, New et al. measured pressures generated in vivo during pressurization and found values of 49 ± 17 kPa and 47 ± 17 kPa for two surgeons.^22^ The results reported in our study are within that range. Noble and Swarts found that the cement pressure required to achieve an optimal cement penetration of 3–5 mm varied significantly with cement brand and bone porosity and therefore there is not an ideal pressure to aim for.^27^ Although it appears pressures were measured at the rim and the pole in a study by Bernowski et al. they do not report figures for the “sustained pressure” but only provide the peak pressure at the rim. Estimating from a provided chart it appears that the sustained pressure at the rim was between 80 and 90 kPa and between 60 and 80 kPa at the pole. This is for an applied load of 201 N. This finding is not reflected in our results where the pressures generated were larger at the pole than the rim. If full contact is assumed between the cup and cement and the difference in the applied load is accounted for, then the magnitude of the pressure appears to be similar to our study. In a chapter on “optimal cementing technique,” Parsch et al. published a graph that report the pressures generated across the acetabular surface using a standard acetabular pressuriser. Although the peak pressures generated were larger than in our study (≈130 kPa), they found no pressure differential during pressurization as was also found in our study.^28^ Ørskov et al. investigated the pressures generated during the pressurization stage of cemented acetabular cup implantation. They applied an 80 N load to a conventional Smith and Nephew pressuriser for 1.5 minutes. They do not report the pressures generates within the acetabulum during pressurization.^23^

The pressuriser effectively seals the acetabulum cavity, and the viscosity of the cement is still sufficiently small so that the pressure is equalized. At the cup implantation stage, there is a flow of excess cement that must be displaced for correct cup positioning. This flow must be driven by a pressure differential. This flow at the cup implantation stage while the cement is curing may be of importance.

### 5.2 Cup insertion

In this study, it was found that the pressure at the pole (0°) in the fourth pentile of cup insertion for non‐vacuum mixed cement was larger for unflanged cups, generating a pressure 14.7% larger than flanged cups. The other location of significant difference between the pressures generated was for the last two pentiles of vacuum mixed cup insertion at 45° from the direction of the applied loading. Unflanged cups generated a pressure 8.76% larger than flanged cups in the fourth pentile and 12.6% larger than flanged cups in the final pentile. In an in vitro experiment, Oh et al. found that a flanged cup produced pressures of 1440 kPa at the pole and 1050 kPa at the rim for flanged acetabular cups, and just 113 kPa at the pole and 73 kPa at the rim for unflanged components. This extreme difference is accounted for by the insertion loads for the cups. A force of 2167 N was used for the flanged component and just 113 N was used for the unflanged cup. There was no justification for this difference in the methodology section, presumably it was due to the instrument being used in position‐control mode rather than load‐control. Those results are therefore not comparable with ours nor are they clinically relevant as no surgeon could maintain a 2 kN force.^16^ A study by Beverland et al. used a similar methodology to our study. A 98.1 N load was applied to the cup using a 10 kg mass. Flanged and unflanged cups were implanted into an irregular mock acetabulum but only the pressure at the pole of the acetabulum was reported. They found an average pressure of 28.4 kPa for flanged components and 41.5 kPa for the unflanged component. The larger pressure for unflanged cup at the pole of the acetabulum can also be seen in our data but the magnitude of the difference was far less significant. They also found that the pressure decayed significantly for each of the cup designs.^15^ This was not found in the our results; this may be because Beverland et al. used a model acetabulum with an irregular rim. Lankester et al. used position and speed‐control for load application and report pressure profiles which reflect the methodology with the force increasing rapidly until peaking at 30 s then quickly decaying to 0 MPa, this is not advisable in vivo as a consistent pressure is required to combat back bleeding.^25^ The addition of a flange increased pressure by a factor of 10 at the rim but by a factor of 2–4 at the pole.^14^ This was not seen in the present study. Parsch et al. performed cadaver experiments with an applied force of 60–100 N. They found that the addition of a flange increased the peak pressure but not the average pressure, the average pressure is a more important measure in cementation to prevent back bleeding. There were only minor differences found between the average pressures generated due to the cup design in the present study. Ørskov et al. investigated the pressures generated during the implantation of flanged and unflanged cemented acetabular cups into a ceramic acetabulum model. During the position‐controlled insertion of the cup, they generated a similar insertion load as was used in this study: 68 N for flanged cups and 57 N for unflanged cups. This load resulted in pressures of 47.1 kPa at the pole and 37.7 kPa at the rim for flanged cups, and 53.6 kPa at the pole and 33.6 kPa at the rim for unflanged cups.

The study reported here is novel in that the methodology included pressurization of the cement prior to cup insertion, thus more closely simulating an in vivo implantation. It is also novel as the whole pressure profile through time was recorded and is reported here, allowing future researchers to refer to this study when a methodology is being designed. Preliminary testing was performed to ensure that the forcing program would not cause “bottoming out” or “flanging out” where some part of the cup comes into direct contact with the acetabulum, preventing further pressurization. Although contact between the acetabulum and the flange was not observed, the cement between the two would have increased the contact surface area between cup and cement, and therefore the pressure generated due to the applied load is reduced. This would account for the unflanged component producing larger pressures.

The function of the flange should not simply be thought of to increase the pressures generated in the acetabulum. With the same applied load and a larger projected area (due to the flange) a smaller pressure should be generated, as reported here. Instead, the flange should be seen as feature to slow the insertion of the cup into the cement by reducing the gap between the acetabular cup and the acetabulum through which cement can flow, in this way the surgeon must apply a larger load in order to correctly position the acetabular cup and therefore produce larger pressures. This may explain the discrepancy often seen between the lower pressures generated by flanged components seen in this study and the improved longevity of flanged acetabular cups observed in vivo.^9^ This explanation is a key outcome of this study.

With some exceptions stated above, this study found that for the most testing conditions, there is always a significant difference between the pressure at the rim and at the pole of the acetabulum during cup insertion (Table 1). As the acetabular cup is inserted, it will create a pressure differential, driving the flow of cement around the cup and out of the acetabulum. As polymerization continues the viscosity of the cement increases, reducing and eventually stopping the flow of cement out of the acetabulum.

There are significant differences between this experimental in vitro study and the clinical in‐vivo setting, however, this study was designed to reduce confounding factors so that concrete conclusions could be drawn. Only one cement was used in this study, more cements should be tested to determine whether the conclusions drawn apply more generally. More work should be done to determine the loading applied in vivo, an instrumented acetabular pressuriser is currently being designed by the authors and further work should be done to determine the force used during cup implantation. During the experiments, measurements of the insertion speed of the cup could have been used to further investigate the effect of the flange on cemented acetabular cup implantation. The outer diameter of the cup used was 50 mm. This is 2 mm larger than should be used for an acetabulum 52 mm in diameter. It is not known what affect the gap between the acetabular cup and the acetabulum has on the pressures generated. Further tests should be performed to determine whether the difference between the diameter of the acetabular cup and the acetabulum makes a significant difference to the pressures generated at the acetabulum surface. The rim of an anatomically correct acetabulum is irregular this would probably lead to larger gaps between the pressuriser and the acetabulum and the cup and the acetabulum. Although the cement penetration was not directly measured it has been shown that penetration is improved with an increased pressure.^10^ If the surface of the acetabulum was porous, then the experiment would be more clinically relevant; however, it would also reduce the accuracy of the pressure transducers used as cement may contact other non‐measuring surfaces of the transducers or a barrier would have to be placed between the cement and the transducers thus altering the pressure data. Five repeats were performed for each testing condition, more repeats would increase the strength of the statistical analysis. In the future work, the thickness of the cement mantles should be measured. When replicating in vivo cemented implantation of the acetabular cup in vitro a choice must be made to use position‐controlled, force‐controlled, or surgeon‐controlled insertion of the cup. Position‐controlled insertion can create forces unattainable during surgery. Force‐controlled insertion can result in incorrect positioning of the cup. And surgeon‐controlled insertion introduces more variables that are uncontrollable between repeats. In this experiment, force control was used to eliminate confounding factors. An experiment analyzing the effects of each of these methodological approaches might be useful. Alternative statistical approaches such as linear regression could be used to analyze the data, this may provide further insights into how the pressure changes though time and use more of the data points collected. However, due to the variation in the time that pressurization commenced, this approach would also have several limitations.

The results suggest that flanged cups provide no advantage in terms of an increase of the pressure differential between rim and pole. Nor does the addition of a flange increase the pressure magnitude compared to unflanged acetabular cups. The data reported here suggest that unflanged cups may produce a larger cement pressure than flanged cups during acetabular cup insertion for the same insertion load.

## 6 CONCLUSION

The results from this study demonstrate that the flange itself does not increase the pressure of cement in the acetabulum but rather, the increased contact area between cup and cement due to the addition of a flange reduces the pressure generated for the same applied load and slows the insertion of the cup. Therefore, to achieve correct positioning in good time, the surgeon will have to apply a larger load to the flanged cup.

## CONFLICT OF INTEREST

The author(s) declared no potential conflicts of interest with respect to the research, authorship, and/or publication of this article.

## FUNDING

The author(s) declared the following potential conflicts of interest with respect to the research, authorship, and/or publication of this article: Alex T. Boote and Philip J. Hyde received research funding from Zimmer Biomet UK Ltd and the EPSRC (DTP studentship from the National Productivity Investment Fund Ref: EP/R512588/1).

## Data Availability

The data that support the findings of this study are available from the corresponding author upon reasonable request.
